# Diffusion kurtosis imaging of gray matter in young adults with autism spectrum disorder

**DOI:** 10.1038/s41598-020-78486-w

**Published:** 2020-12-08

**Authors:** Faye McKenna, Laura Miles, Jeffrey Donaldson, F. Xavier Castellanos, Mariana Lazar

**Affiliations:** 1 Department of Radiology, Center for Biomedical Imaging, New York University School of Medicine, 660 First Ave, Fourth Floor, New York, NY USA; 2 Department of Child and Adolescent Psychiatry, New York University School of Medicine, New York, NY USA; 3 Vilcek Institute of Graduate Biomedical Sciences, New York University School of Medicine, New York, NY USA; 4 Nathan Kline Institute for Psychiatric Research, Orangeburg, NY USA

**Keywords:** Autism spectrum disorders, Autism spectrum disorders, Diffusion tensor imaging

## Abstract

Prior ex vivo histological postmortem studies of autism spectrum disorder (ASD) have shown gray matter microstructural abnormalities, however, in vivo examination of gray matter microstructure in ASD has remained scarce due to the relative lack of non-invasive methods to assess it. The aim of this work was to evaluate the feasibility of employing diffusional kurtosis imaging (DKI) to describe gray matter abnormalities in ASD in vivo. DKI data were examined for 16 male participants with a diagnosis of ASD and IQ>80 and 17 age- and IQ-matched male typically developing (TD) young adults 18–25 years old. Mean (MK), axial (AK), radial (RK) kurtosis and mean diffusivity (MD) metrics were calculated for lobar and sub-lobar regions of interest. Significantly decreased MK, RK, and MD were found in ASD compared to TD participants in the frontal and temporal lobes and several sub-lobar regions previously associated with ASD pathology. In ASD participants, decreased kurtosis in gray matter ROIs correlated with increased repetitive and restricted behaviors and poor social interaction symptoms. Decreased kurtosis in ASD may reflect a pathology associated with a less restrictive microstructural environment such as decreased neuronal density and size, atypically sized cortical columns, or limited dendritic arborizations.

## Introduction

Autism spectrum disorder (ASD) is a neurodevelopmental disorder characterized by a range of social, executive functioning, and sensory deficits^1^, with a prevalence as high as 1 in 50 children^2^. Individuals with ASD have been found to have an array of complex brain abnormalities including early overgrowth^3^, microstructural disorganization^4^, and deficits in both cytoarchitecture^5^ and functional connectivity^6^. In vivo studies of microstructural abnormalities in ASD have focused primarily on white matter^7,8^, leaving the nature and distribution of gray matter (GM) abnormalities largely uncharacterized.

Postmortem studies of ASD have implicated several abnormalities in cortical GM. Early studies found increased cell packing, particularly in the prefrontal cortex^4,9^ and decreased cell size in the cingulate cortex^10^ and frontal lobe regions^4^, along with heterotopia and decreased dendritic branching in frontal and temporal gyri^4,11^. Ex vivo research has also shown increased markers of inflammation in the anterior cingulate cortex and middle frontal gyrus^12^, along with under-developed minicolumn formation across the cortex^5,13–15^. Although ex vivo studies have been essential for understanding the underlying cellular pathology of ASD, the literature is notably variable likely due to the heterogeneous nature of the disease and the inevitable limitations of postmortem research, which is often based on highly inhomogeneous and small samples ranging from 1 to 11 ASD subjects^4,5,10,12–17^, and typically focuses on isolated brain regions. Thus, techniques for examining GM microstructure in vivo would allow whole-brain analysis within larger, well-matched samples. Such techniques are necessary for better describing the location, degree and developmental course of GM microstructural pathology and their variation across individuals affected with ASD.

Diffusion-weighted MRI (dMRI) is a powerful tool for describing microstructural tissue organization at micrometer scale by probing water molecule displacement, which is sensitive to microscopical barriers (e.g., membranes) and organelle organization (e.g., density, orientation) within each voxel. To date, diffusion tensor imaging (DTI), which approximates water diffusion in tissue using a Gaussian distribution, has remained the most widely used diffusion technique. DTI associated indices, fractional anisotropy (FA), and mean (MD), axial (AD), and radial (RD) diffusivities have been widely applied to characterize anisotropic white matter and have been found to be sensitive to a number of white matter pathological changes such as dysmyelination, axonal loss, and inflammation^18–21^. The advent of high angular resolution multi-shell diffusion imaging methods has allowed extending the in vivo investigation of microstructure to gray matter^22,23^. Among these new methods, diffusional kurtosis imaging (DKI) was the first to show high sensitivity to GM changes due to development in typical children and adolescents as well as to document atypical development in attention-deficit hyperactivity disorder (ADHD), a childhood disease^24^. DKI improves upon the DTI characterization of the diffusion signal in tissues by including an additional directional metric, the kurtosis index, to describe non-gaussian diffusion contributions to the MR signal^25^. Non-Gaussian diffusion contributions are inherent to biological tissues such as the brain and arise from the complex organization of microscopic membranes, organelles, and neuronal compartments^25^. Of note, kurtosis metrics have been shown to be able to describe complex structure in the absence of anisotropy^25,26^. Increased kurtosis is assumed to describe increasingly restricted and complex environments and has been shown to characterize brain development and maturation^23,24^, as well as pathological features leading to increased complexity (e.g., astrogliosis or microgliosis)^27–29^. Decreased kurtosis is likely indicative of tissue disintegration and shrinkage^30–32^. DKI provides both a mean kurtosis (MK) metric, which characterizes kurtosis averaged across all spatial directions, and, like DTI, two directional metrics: axial kurtosis (AK), which quantifies kurtosis along the primary or largest diffusivity direction and radial kurtosis (RK), which measures the averaged kurtosis in the plane perpendicular to the primary diffusivity direction. The use of directional kurtosis metrics in GM is supported by recent findings indicating highly directional diffusion in the cortex aligning either radially or both radially and tangentially to the cortical ribbon^22,33^.

To date, DKI has been shown to be sensitive to GM microstructural change in the aging brain^34–36^ and in several conditions including Parkinson’s disease^37^, chronic pain^38^, multiple sclerosis^39^, traumatic brain injury^40^, multiple sclerosis^39^, schizophrenia^41^, Alzheimer’s disease^42^ and attention-deficit/hyperactivity disorder^43^. Ex vivo studies investigating GM changes in several rodent neurodegenerative models have documented that increased kurtosis parallels increased fiber dispersion^29^, protein accumulation^27^, and astrogliosis^28^, and that decreased kurtosis co-occurs with cytotoxic edema^44^, abnormal neurofilaments, and axonal shrinkage and loss^31,45^. In addition, directional kurtosis estimates have been found to detect unique characteristics of tissue compared to MK in both healthy and clinical samples^35,40,46^. Therefore, we hypothesized that mean and directional kurtosis in the GM of ASD patients is altered as a result of one or more cellular pathological processes observed in ASD: disorganization and alterations of cortical laminar cytoarchitecture and dendrites, decreased cell size, increased cell packing, gliosis, and narrow minicolumn formation^4,13,17,47^.

To date, only few studies have investigated GM cortical microstructure in ASD using in vivo dMRI methods^48–50^. An initial study found slightly increased MD in total brain GM in adolescents with ASD ages 12–18 years old^48^, while another study employed a Restriction Spectrum Imaging (RSI) approach and reported significantly reduced gray matter neurite density (ND) in the left fusiform cortex of adolescents with ASD ages 7–17 years old at multiple-comparison correction level^49^. This second study additionally found slightly increased MD in the parietal and occipital lobes, but this finding did not survive multiple-comparison correction^49^. Most recently, reduced orientation dispersion (ODI) and ND were found in ASD patients with impaired recognition of facial emotional expressions in the occipital lobe, fusiform gyrus, and inferior parietal and superior temporal regions using the Neurite Orientation and Dispersion Imaging (NODDI) approach^50^. Reduced ND in ASD was hypothesized to be related to the ex vivo finding of a lower density of, or thinning of, myelinated axons in the gray matter cortex, while decreased ODI was postulated to be associated with the loss of dendritic spines^48,49^. The increased MD noted in two of the studies was proposed to be a result of increased water due to the inflammatory response often reported in ASD^48,49^. This previous work has suggested that dMRI may be able to distinguish different GM microstructural pathologies in ASD.

In this study, we employed DKI to characterize, non-invasively and in vivo*,* gray matter cortical differences in a group of young adults with ASD versus age, sex and IQ-matched typically developing (TD) control participants and to test the hypothesis that kurtosis is associated with the severity of core ASD symptom deficits (i.e., communication, repetitive behaviors, and social interaction). Compared to biological model-based multi-shell dMRI methods, such as NODDI, DKI is a data driven mathematical model that makes no assumptions regarding underlying microstructure, and thus is independent of such assumptions, which may not be valid in various pathologies. Moreover, compared to DTI metrics, DKI metrics have been shown to be robust with respect to partial volume averaging with cerebro-spinal fluid^51^, an artifact likely to at least partially affect cortical regions.

## Results

### Participants

Demographic characteristics for both groups are presented in Table 1. There were no group differences in age (*p* = 0.678), education (*p* = 0.463), or full-scale IQ score (*p* = 0.143) (Table 1).

**Table 1:** Summary of demographic, IQ, ADIR, and ADOS severity scores of the participants in TD and ASD groups.

	Autism spectrum disorder (*n* = 16)	Typically developing (*n* = 17)	t-test *p* value
Male/female	16/0	17/0	–
Handedness (right/left)	15/1	16/1	.809
Age (years) Mean ± SD	21.4 ± 2.4	21.7 ± 2.1	.678
Range	18–25	18–24	
Education (years) Mean ± SD	14.90 ± 1.70	15.3 ± 1.5	.463
Range	12–18	13–17	
WAIS-III FSIQ Mean ± SD	108.88 ± 17.39	116.65 ± 11.98	.143
Range	85–148	98–143	
ADI-R RRB Mean ± SD	5.55 ± 1.75	–	–
Range	3–9		
ADI-R COM Mean ± SD	15.27 ± 5.0	–	–
Range	9–24		
ADI-R SOC Mean ± SD	19.18 ± 7.0	–	–
Range	6–30		
ADOS SOC Mean ± SD	3.88 ± 1.6	–	–
Range	1–6		
ADOS COM Mean ± SD	1.38 ± 0.7	–	–
Range	1–3		
ADOS COM SOC Mean ± SD	3.88 ± 1.5	–	–
Range	1–6		
ADOS SARRB Mean ± SD	5.31 ± 2.1	–	–
Range	1–7		
ADOS SBRI Mean ± SD	1.06 ± 0.3	–	–
Range	1–2		
ADOS SA Mean ± SD	4.31 ± 2.2	–	–
Range	1–8		

*ADIR* Autism Diagnostic Interview-Revised, *ADOS* Autism Diagnostic Observation Schedule, *FSIQ* Full scale intelligence quotient, *RRB* Restrictive & repetitive behaviors, *COM* Communication, *SOC* Social Interaction, *COM SOC* Communication + Social Interaction, *SARRB* Social Affect & Restricted Repetitive Behaviors, *SA* social affect, *SBRI* Stereotyped behaviors and restricted interests.

### Between-group comparisons

**Figure 1:**
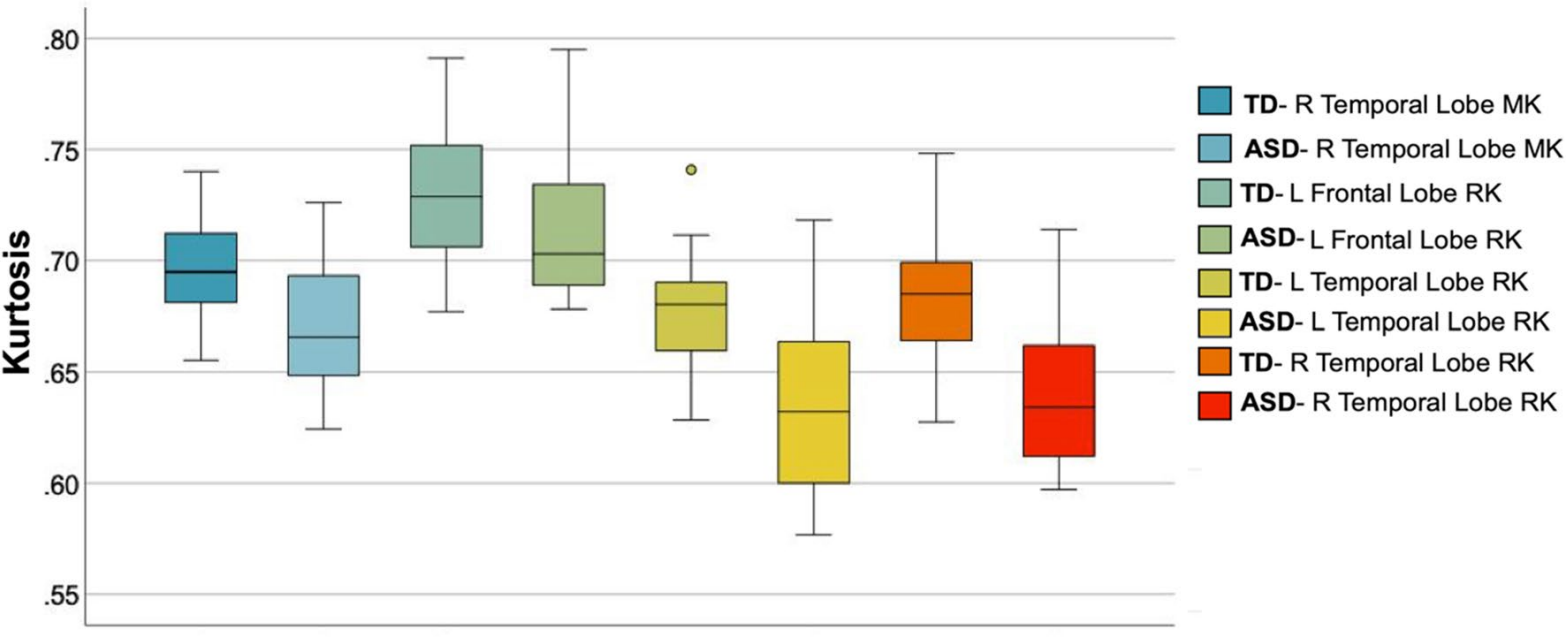
Decreased MK and RK in the right and left temporal lobe GM, and decreased RK in the left frontal lobe GM are observed in autism spectrum disorder compared to the typically developing group. All results shown were significant after ANCOVA between-group tests controlling for age and correcting for multiple comparisons (q ≤ .05 BH FDR).

At the lobar level, individuals with ASD demonstrated significantly lower RK in the left frontal, and right and left temporal lobe GM, and significantly lower MK in the right temporal lobe GM at the q ≤ 0.05 BH FDR-corrected cut-off^52^ (Table 2; Fig. 1).

**Table 2:** Differences in diffusion metrics (MK, RK and MD) in autism spectrum disorder compared to typical developing young adults in the frontal, temporal and parietal lobes and sub-lobar gray matter regions-of-interest in the brain revealed through ANCOVA analysis controlling for age.

Region	R/L	MK ASD	MK TD	ANCOVA *p* value*s* MK	MK Cohen’s d	RK ASD	RK TD	ANCOVA *p* value*s* RK	RK Cohen’s d	MD ASD (µm^2^/ms)	MD TD (µm^2^/ms)	ANCOVA *p* value*s* MD	MD Cohen’s d
**Frontal lobe**													
Frontal lobe	Right	.70 ± .02	.72 ± .03	.039	.232	.71 ± .03	.73 ± .03	.028	.395	1.18 ± .09	1.25 ± .07	.030	.408
Frontal lobe	Left	.71 ± .02	.72 ± .03	.034	.271	.71 ± .02	.74 ± .03	.011*	.462	–	–	–	–
Caudal middle frontal	Left	.73 ± .03	.76 ± .04	.038	.391	.74 ± .03	.79 ± .05	.020	.499	–	–	–	–
Lateral orbitofrontal	Right	.66 ± .04	.68 ± .03	.001*	.418	.63 ± .05	.67 ± .04	.002*	.447	–	–	–	–
Lateral orbitofrontal	Left	.67 ± .03	.68 ± .03	.010	.179	.64 ± .04	.66 ± .04	.011	.205	–	–	–	–
Paracentral	Right	.71 ± .05	.72 ± .05	.022	.031	–	–	–	–	1.21 ± .13	1.31 ± .11	.029	.409
Pars opercularis	Right	.71 ± .03	.72 ± .03	.004*	.302	.72 ± .03	.75 ± .04	.014	.432	–	–	–	–
Pars opercularis	Left	.71 ± .03	.73 ± .03	.004*	.398	.72 ± .03	.76 ± .03	< .001*	.561	–	–	–	–
Pars triangularis	Right	.73 ± .31	.74 ± .04	.025	.144	.73 ± .04	.76 ± .05	.034	.309	1.14 ± .09	1.19 ± .05	.047	.381
Precentral	Left	–	–	–	–	–	–	–	–	1.25 ± .09	1.32 ± .10	.050	.372
Rostral middle frontal	Left	–	–	–	–	.71 ± .02	.73 ± .04	.017	.425	–	–	–	–
Superior frontal	Right	–	–	–	–	.68 ± .03	.70 ± .05	.022	.253	1.19 ± .09	1.28 ± .09	.007	.516
Superior frontal	Left	.69 ± .03	.71 ± .03	.035	.251	.69 ± .04	.72 ± .04	.002*	.412	–	–	–	–
**Parietal lobe**													
Parietal lobe	Right	–	–	–	–	–	–	–	–	1.20 ± .07	1.27 ± .1	.039	.395
Parietal lobe	Left	–	–	–	–	.74 ± .03	76 ± .03	.039	.346	–	–	–	
Inferior parietal	Right	–	–	–	–	.74 ± .03	.76 ± .03	.047	.287	–	–	–	–
Precuneus	Right	.68 ± .04	.70 ± .03	.003*	.327	.69 ± .05	.72 ± .04	.005*	.307	–	–	–	–
Precuneus	Left	.68 ± .03	.71 ± .03	.029	.330	.69 ± .04	.72 ± .03	.001*	.441	–	–	–	–
Superior parietal	Right	–	–	–	–	–	–	–	–	1.3 ± .10	1.4 ± .15	.022	.442
Supramarginal	Right	–	–	–	–	.72 ± .04	.75 ± .03	.023	.454	–	–	–	–
**Temporal lobe**													
Temporal lobe	Right	.67 ± .03	.70 ± .02	.005*	.547	.64 ± .04	.69 ± .03	< .001*	.694	–	–	–	–
Temporal lobe	Left	–	–	–	–	.64 ± .04	.68 ± .03	.002*	.622	1.09 ± .05	1.12 ± .04	.040	.280
Bankssts	Right	.72 ± .03	.75 ± .04	.031	.443	.73 ± .04	.77 ± .05	.017	.452	–	–	–	–
Fusiform	Right	–	–	–	–	.63 ± .06	.67 ± .04	.018	.460	–	–	–	–
Parahippocampal	Right	–	–	–	–	.62 ± .07	.66 ± .04	.021	.344	–	–	–	–
Parahippocampal	Left	–	–	–	–	.59 ± .06	.65 ± .05	.024	.477	–	–	–	–
Inferior temporal	Right	–	–	–	–	.58 ± .04	.63 ± .05	.015	.552	–	–	–	–
Middle temporal	Right	–	–	–	–	.64 ± .04	.68 ± .04	.019	.503	1.02 ± .04	1.06 ± .06	.042	.386
Middle temporal	Left	.65 ± .04	.69 ± .03	.027	.509	.63 ± .04	.67 ± .04	.011	.572	–	–	–	–
Superior temporal	Right	.70 ± .03	.72 ± .03	.015	.486	.69 ± .03	.73 ± .03	.001*	.660	1.14 ± .06	1.23 ± .12	.008	.531
Superior temporal	Left	.69 ± .03	.72 ± .02	.004*	.637	.68 ± .03	.72 ± .03	.003*	.663	–	–	–	–
Temporal pole	Right	–	–	–	–	–	–	–	–	1.16 ± .14	1.30 ± .15	.014	.457
**Other**													
Insula	Right	.66 ± .04	.68 ± .03	.049	.374			–	–	–	–	–	–
Insula	Left	.65 ± .03	.68 ± .03	.003*	.492	.67 ± .03	.71 ± .04	.002*	.528	–	–	–	–
Isthmus cingulate	Right	.64 ± .06	.67 ± .06	.014	.224	.65 ± .08	.68 ± .08	.035	.160	–	–	–	–
Isthmus cingulate	Left	.65 ± .60	.68 ± .05	.015	.348	.66 ± .08	.69 ± .06	.040	.275	–	–	–	–
Caudal anterior cingulate	Right	–	–	–	–	–	–	–	–	1.06 ± .07	1.12 ± .10	.046	.378
Posterior cingulate	Right	.63 ± .05	.67 ± .05	.009	.421	.64 ± .07	.69 ± .08	.029	.317	1.03 ± .07	1.12 ± .07	.001*	.644
Posterior cingulate	Left	.65 ± .04	.69 ± .06	.007	.338	.67 ± .06	.71 ± .06	.013	.347	1.07 ± .08	1.13 ± .09	.046	.358
Rostral anterior cingulate	Right	.59 ± .04	.62 ± .05	.044	.404	–	–	–	–	–	–	–	–
Rostral anterior cingulate	Left	.60 ± .04	.63 ± .05	.013	.268	.60 ± .04	.63 ± .06	.028	.287	–	–	–	–

All tests shown reach trend-level at *p* ≤ 0.05, uncorrected; tests that remain significant after the BH procedure for multiple comparisons correction at q $\le$ 0.05 are indicated by an asterisk (*) along with the effect size (Cohen’s d).

**Figure 2:**
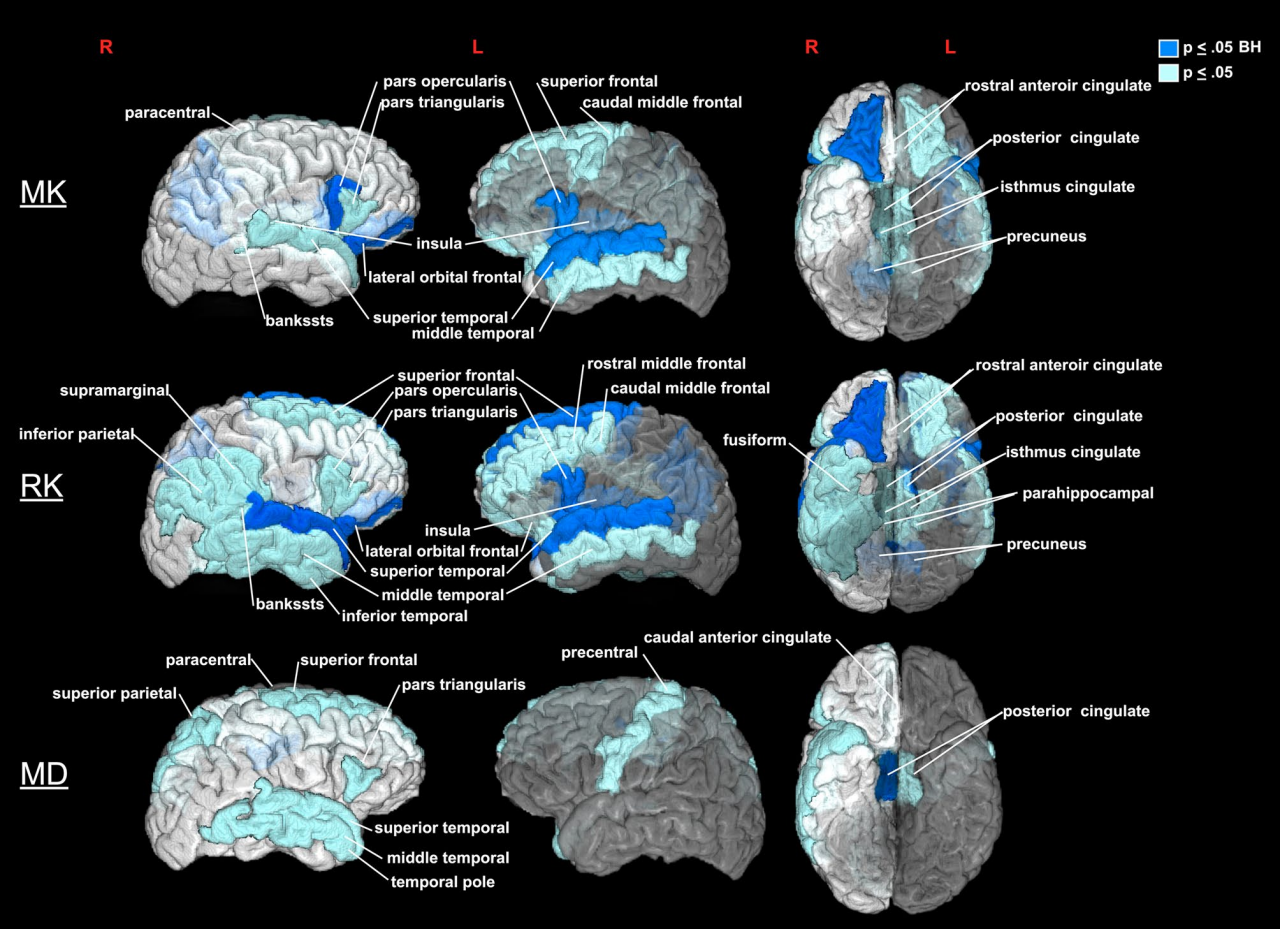
Brain areas with decreased diffusion metrics (MK, RK and MD) in young males with autism spectrum disorder compared to a typically developing group. The color reflects the significance level (*p* ≤ .05, uncorrected (light blue) and q ≤ .05 BH corrected (dark blue). Solid colors are used for outer brain surface areas with see-through regions depicted by transparent colors.

At the sub-lobar level, RK demonstrated the most robust between-group differences with a significant decrease in the ASD group noted in the left pars opercularis division of the inferior frontal gyrus, left superior frontal and bilateral superior temporal gyri, right lateral orbital frontal, bilateral precuneus and left insular cortex regions of interest (ROIs) (Table 2; Fig. 2). Significantly decreased MK in ASD was found in the bilateral pars opercularis division of the inferior frontal gyrus, left superior temporal gyri, right lateral orbital frontal, right precuneus and left insular cortices ROIs (Table 2; Fig. 2). Between-group MD comparisons revealed fewer yet similar regions of decreased diffusivity in ASD compared to TD participants shown through analyses of RK and MK with only the right posterior division of the cingulate cortex reaching significance after multiple comparison corrections.

No differences in AK were found between groups in either lobar or sub-lobar regions.

### Association with disease severity

**Figure 3:**
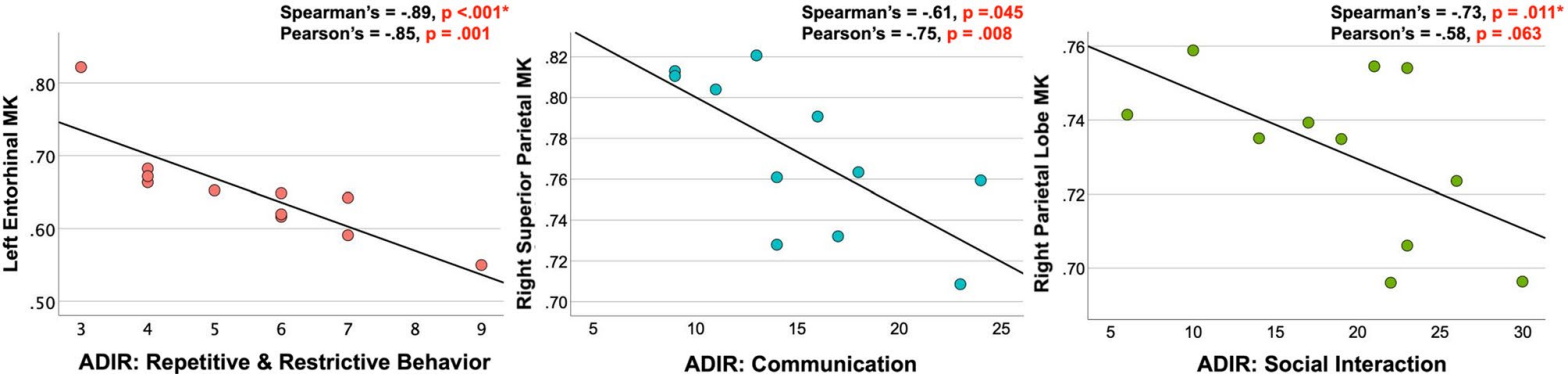
Brain areas with significant associations between performance on the 3 sub-categories of the ADI-R and mean kurtosis in the ASD group. A lower score on the ADI-R indicates less severe symptoms and is associated with increased MK in ASD. The strength of the correlation and the corresponding *p* value are listed for both Spearman’s and Pearson’s tests for each graph.

In the ASD group, increased MK was associated with fewer repetitive and restrictive behaviors and better social interaction, with only the left entorhinal cortex’s association with repetitive and restrictive behaviors and the right parietal lobe’s association with social interaction reaching significance after correction for multiple comparisons (Table 3; Fig. 3). At trend-level and to a lesser extent, increased RK and MD were correlated to less restrictive and repetitive behaviors and increased RK and MD were correlated to better communication (Supplementary Tables 1 and 2).

**Table 3:** Spearman’s and Pearson’s correlations assessing associations between MK and lifetime clinical symptoms as measured by ADI-R.

MK ROI	Hemisphere	Test	ADI-R restrictive &repetitive behaviors r/p	ADI-R communication r/p	ADI-R social interaction r/p
Medial orbitofrontal	Right	Spearman’s	− .73/.011	–	− .66/.028
		Pearson’s	− .73/.010	–	− .67/.022
Medial orbitofrontal	Left	Spearman’s	− .68/.020	–	− .61/.048
		Pearson’s	− .63/.039	–	–
Paracentral	Left	Spearman’s	–	–	− .75/.008
		Pearson’s	–	–	− .663/.026
Pars opercularis	Left	Spearman’s	− .67/.025	–	− .62/.042
		Pearson’s	− .69/.020	–	–
Pars triangularis	Left	Spearman’s	− .67/.025	–	− .73/.011
		Pearson’s	–		–
Parietal lobe	Right	Spearman’s	–	–	− .73/.011*
		Pearson’s	–	–	–
Inferior parietal	Right	Spearman’s	–	–	− .73/.010
		Pearson’s	–	–	–
Precuneus	Left	Spearman’s	− .67/.024	–	− .74/.009
		Pearson’s	− .70/.016	–	− .68/.022
Superior parietal	Right	Spearman’s	–	− .61/.045	–
		Pearson’s	–	− .75/.008	–
Superior parietal	Left	Spearman’s	–	− .69/.019	–
		Pearson’s	–	–	–
Supramarginal	Right	Spearman’s	− .62/.042	–	–
		Pearson’s	–	–	–
Entorhinal	Left	Spearman’s	− .89/ < .001*	–	− .67/.025
		Pearson’s	− .85/.001	–	–
Parahippocampal	Left	Spearman’s	–	–	− .76/.007
		Pearson’s	–	–	–
Insula	Left	Spearman’s	–	–	− .68/.020
		Pearson’s	–	–	–
Isthmus cingulate	Left	Spearman’s	− .67/.025	–	− .73/.012
		Pearson’s	− .683/.02	–	–
Posterior cingulate	Right	Spearman’s	− .71/.015	–	− .68/.023
		Pearson’s	− .77/.006	–	− .66/.026
Posterior cingulate	Left	Spearman’s	− .61/.049	–	–
		Pearson’s	–	–	–
Rostral anterior cingulate	Right	Spearman’s	–	–	− .65/.030
		Pearson’s	–	–	–

A higher score on the ADI-R indicates increased severity. All tests shown reached trend-level at *p* ≤ 0.05, uncorrected. Tests that remain significant after the Benjamini–Hochberg correction for multiple comparisons are indicated by an asterisk (*) (q ≤ .05 BH FDR).

## Discussion

The data presented here provides, for the first time, in vivo evidence of GM microstructural pathology in ASD by employing DKI to non-invasively probe tissue organization and complexity. Significantly decreased cortical kurtosis and diffusion noted in the ASD group may reflect one or several pathological processes reported by previous histological studies. The majority of ex vivo studies on ASD found decreased cell size and limited dendritic arbors^17^, while narrower cell minicolumns with decreased neuropil in the GM of patients with ASD have been extensively documented^5,13,16^. These findings suggest reduced microstructural complexity in the GM of the ASD brain, consistent with our findings of decreased MK and RK. Further supporting this hypothesis, rodent studies have reported that decreases in MK may reflect neuronal loss, microgliosis, myelin disruption^31^, neuron disorganization and cytotoxic edema^44^, with decreases in both MK and RK reflecting reduced GM neurite density^53^.

**Figure 4:**
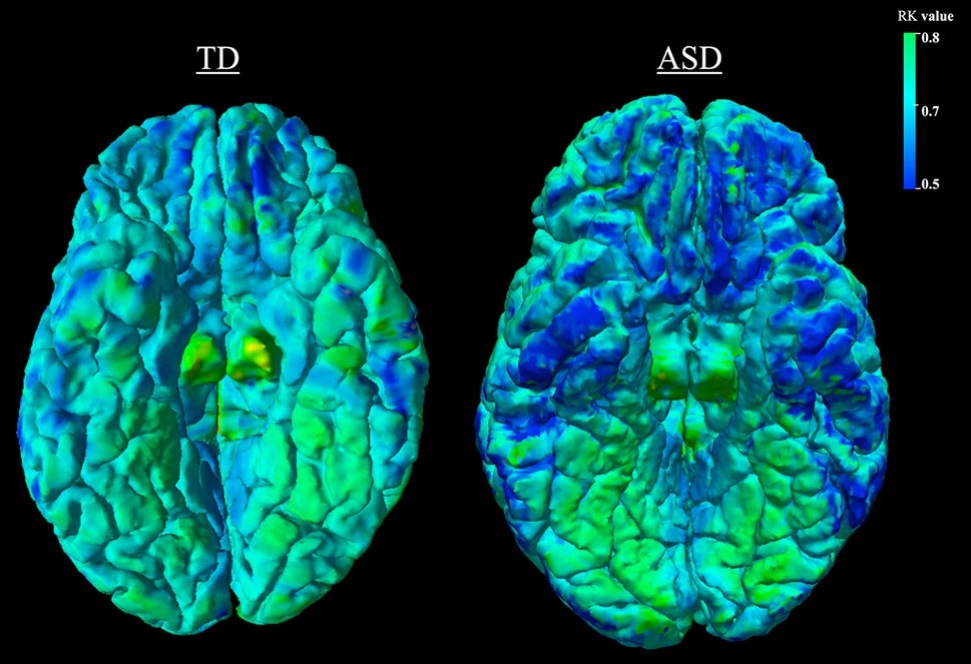
Surface map of radial kurtosis values in a typically developing versus an autism spectrum disorder participant from an inferior view of the temporal, frontal and cingulate areas. RK values are projected from a middle GM cortical layer depth. Dark blue indicates lower values, while bright green indicates higher values.

MK, RK, and MD measure different characteristics of water diffusion, and are likely sensitive to somewhat different microstructural abnormalities in GM tissue^25^. In the data investigated here, RK, a DKI-specific parameter of diffusional kurtosis perpendicular to the primary direction of diffusion, was the most sensitive metric in quantifying between-group differences, highlighting areas with known cellular pathology in ASD (Table 2; Figs. 2 and 4). The decreased MK in ASD was primarily driven by decreases in RK, with AK showing no quantifiable between-group differences. MD, a classic measure of Gaussian diffusion, was less sensitive in detecting GM pathology in ASD, therefore highlighting non-Gaussian kurtosis metrics as more powerful in detecting GM microstructural changes.

**Figure 5:**
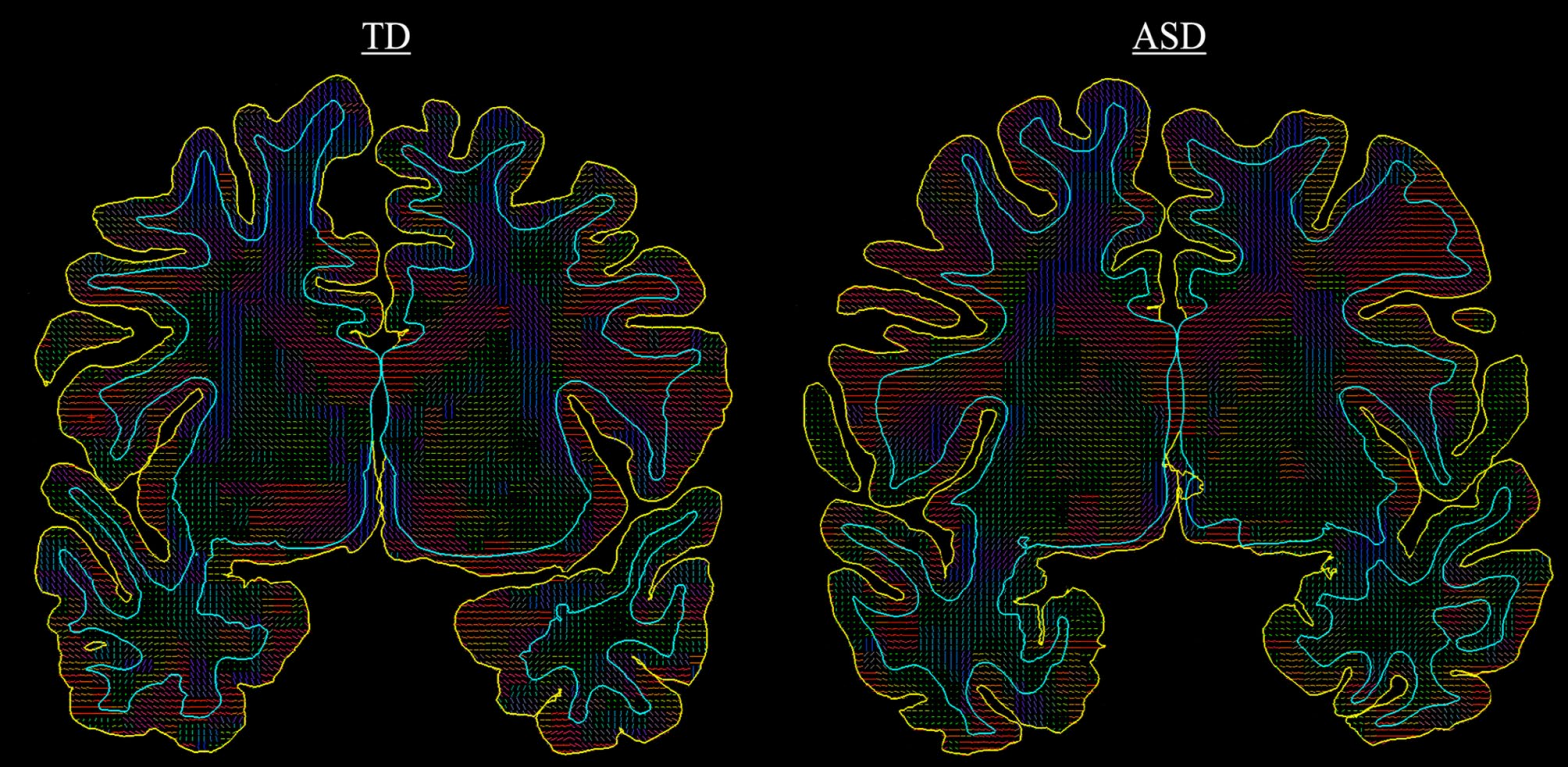
Example of directional diffusion tensors in a mid-brain slice of a typically developing versus autism spectrum disorder participant. Red lines indicate left to right diffusion, blue lines indicate up to down diffusion, and green lines indicate front to back diffusion in the brain. The primary direction of GM diffusion in both brains is largely seen as perpendicular to the cortical mantle (AK), and thus the secondary (radial) diffusion direction is parallel to the cortical mantle (RK).

Overall, our data suggest that microstructural changes in ASD are directional and are likely due to alterations in tissue and/or cellular processes affecting diffusion in the plane orthogonal to the primary diffusion direction. Several studies have found that diffusion tensor orientation in GM is closely related to the orientation of neuronal components such as dendritic and axonal fascicles and glial processes^54–56^. Examination of our data (Fig. 5) as well as previous literature shows that the primary direction of diffusion in the cortical mantle, particularly in gyral regions, is perpendicular to cortical mantle^33,57–59^.

This would suggest that radial kurtosis reflects diffusion changes occurring tangential to the cortical mantle. A noteworthy parallel to our finding is the histological documentation of narrower minicolumns with reduced neuropil, which is comprised of dendrites and ascending/descending axons, in frontal and temporal GM regions in patients with ASD^5,15^. These microscopic columns are aligned perpendicular to the cortical mantle, with narrower unit cytoarchitecture and reduced dendritic processes hypothetically creating less restrictive diffusion tangential to the cortical mantle. Consistent with this hypothesis, our data shows decreased radial kurtosis, i.e., decreased kurtosis perpendicular to minicolumns’ direction. In summary, although multiple cellular processes likely contribute in varying degrees to the direction and restriction of diffusion, our findings of reduced radial kurtosis may be driven by the abnormal minicolumn formation previously documented in histological studies.

Compared with the recent dMRI work investigating GM cortical microstructure in ASD using other methods than DKI^48–50^, our findings of significantly decreased kurtosis in the bilateral temporal lobes, precuneus and superior temporal regions generally align with reported findings of decreased neurite density in temporal and parietal lobe ROIs. Decreased DKI was found to be related to decreased neurite density in a preclinical model^53^. In this study, the DKI approach additionally suggests reduced complexity in the frontal lobe and the insular and cingulate cortex areas. More work will be necessary to explore how different dMRI models (e.g. DKI, NODDI, and RSI) compare in identifying GM microstructural pathology in ASD across the lifespan. The slightly increased MD values in total brain GM and in the parietal and occipital lobes reported by these previous studies, which however did not pass multiple corrections level, are somewhat contradictory to our findings of significantly decreased MD in the right posterior cingulate cortex^48,49^. More research will be important to elucidate where and to what extend MD is altered in varying ASD populations.

The regions found here to have abnormal microstructure in the ASD group are consistent with both neuroimaging and postmortem neuropathology studies, which highlighted the limbic, frontal and temporal cortical regions as being primarily affected by the disorder^12,17,60–65^ (Table 2; Figs. 1 and 2). Altogether, neuroimaging and cognition studies have suggested that higher-order regions are the most affected cortical regions in ASD, possibly because they are slower maturing and therefore have a longer window of vulnerability to pathological disruptions^47^. Additionally, areas involved in social cognition, such as the inferior frontal gyri, and orbitofrontal, medial prefrontal, posterior parietal, cingulate, superior temporal and insular cortices^65,66^, have been key areas of study in ASD due to their role in social interaction and communication deficits, which are among the disorder’s core features^67^. Here we show significant microstructural abnormalities in ASD compared to TD in the pars opercularis division of the inferior frontal and superior frontal gyri, lateral orbital frontal, superior temporal, precuneus, insular and posterior cingulate cortices (Table 2; Fig. 2). Disrupted GM cytoarchitecture of regions within the social-emotional brain network may underlie functional hypoactivation noted using fMRI in an array of tasks including face processing^68^, theory of mind^69^, motion^70^ and self-referential empathy^71^. Thus, DKI mapping of microstructural pathology in these areas, alongside ex vivo and cognition studies, may better explain the core ASD symptoms of social-emotional impairment that originate from these regions^65^.

Reduced symptom severity in the restrictive and repetitive behaviors subcategory of the Autism Diagnostic Interview-Revised (ADI-R)^72^ was significantly correlated with increased MK in the entorhinal cortex, and reduced symptom severity in the social interaction subcategory of the ADI-R was significantly correlated with increased MK in the right parietal lobe within the ASD group (Table 3; Fig. 3). Increased kurtosis in GM and its associated cellular processes may have a protective function or supporting effect, with increased MK in GM correlating to better performance on executive function tests in traumatic brain injury, multiple sclerosis and schizophrenia^39,41,73^. In this study, elevated kurtosis values in ASD, closer to the TD range, appear to be associated with fewer restrictive and repetitive behaviors and better social interaction skills.

Postmortem studies have provided valuable information regarding the underlying GM microstructural pathologies of ASD, most notably reporting increased cell packing^4,9^, decreased cell size and dendritic branching^4,10^ and under-developed minicolumn formation^5,13–15^. However, they are inherently limited by their invasive nature and their findings are difficult to translate into clinical practice and personalized medicine. Advanced dMRI imaging, which is non-invasive and can be acquired in a clinically feasible time frame and setting, may be used to extend and refine these decades-long documented microstructural abnormalities in ASD. Of note, dMRI has mostly been applied to detect in vivo ASD white matter pathologies despite GM showing a larger range of histological abnormalities that may be useful clinical biomarker targets. For example, reduced synaptic density in GM has recently been used as a radiopharmaceutical target in schizophrenia^74^. Kurtosis is an index of microstructural complexity, which in addition to potentially detecting an array of histological changes in ASD it has both successfully revealed the presence of isolated microstructural changes in preclinical models^27,75^ and been superior to conventional macrostructural MRI in differentiating patients in other neurological disorders^37,41^. Therefore, although this pilot study primarily aimed to test the ability of DKI to detect GM microstructural abnormalities in ASD and their relationship to symptoms, its results provide support for future larger DKI studies that may better track, predict and treat ASD deficits.

Several limitations of this study need to be considered. First, the sample size was relatively small, and the study had a purely cross-sectional design. In part, our limited sample size stems from our decision to select a relatively homogeneous group by studying males with ASD within a limited age range (18–25 years old) and without intellectual disability (IQ < 80), which we believe strengthened our ability to detect group differences. However, it will be important to replicate these results in larger samples and to examine gray matter in both sexes and across larger age ranges. Second, these data were acquired before the availability of higher resolution diffusion methods that may reveal more accurate diffusion measurements. Third, two different T1-weighted (T1w) MRI protocols were used for the within-subject atlas segmentation and ROI analysis. Paired t-tests showed that different T1w acquisition had no significant effect on the calculation of MK or RK metrics but had some minimal effects on MD metrics and therefore the MD results presented here should be interpreted cautiously (Supplementary Table 3). Finally, given our relatively small sample size, we present both results surviving multiple comparison correction as well as uncorrected results in the tables and supplementary material which may provide the basis for subsequent attempted replications. The study of GM microstructure in ASD is relatively new and we believe the data presented here should encourage further research on the GM substrates of this disorder’s clinical manifestations.

In conclusion, we report decreased gray matter kurtosis, primarily arising from altered radial kurtosis, in ASD in the frontal and temporal lobes and in a number of functionally distinct cortical ROIs involved in social and emotional processing. Incorporating in vivo metrics of GM microstructure, such as MK and RK, into models of ASD pathology may be instrumental in better characterizing ASD subtypes and their developmental progressions.

## Methods

### Participants

A total of 26 male individuals with a potential ASD diagnosis and 36 age-matched male TD participants were recruited by advertisement within the community and through autism organizations in the New York City metropolitan area. Diagnoses were confirmed by administering the Autism Diagnostic Observation Schedule (ADOS)^76^ to all ASD participants under the supervision of a certified licensed clinical psychologist. Additionally the ADI-R^72^ was administered to the ASD parents who participated in the study. TD participants who reported no personal or family history of ASD or other psychiatric disorders were retained in the study. Participants were excluded for a history of head trauma, organic brain disorder, IQ < 80, and MRI contraindications. The Weschler Adult Intelligence scale (WAIS-III)^77^ was administered to obtain Full Scale intellectual quotient (FSIQ) scores and confirm the lack of intellectual disability. Handedness was obtained using the Chapman and Chapman handedness questionnaire^78^. The study was approved by the institutional review board at the NYU School of Medicine and performed in accordance with all National Institute of Mental Health (NIMH) and NYU School of Medicine relevant guidelines and regulations. For this study, 17 ASD patients and their parents provided informed consent. For another 9 ASD participants, for whom parents were not available for participation, competence to provide consent was assessed at the beginning of the visit by trained personnel. All these study participants were adults deemed able to provide informed consent and provided informed consent before participation according to our institutional rules.

Subjects whose imaging data displayed significant motion artifacts and for whom repeated data acquisition was not successful were not included in the study. A neuroradiologist examined all scans for gross brain abnormalities and to exclude any subjects suspected of an organic brain disorder.

To match the two groups on IQ, TD subjects underwent first only a review of medical history and the IQ assessment. As new ASD participants were added to the study, TD participants were invited for imaging if they had IQ values similar to the recruited ASD participants. Twelve TD participants were either no longer available to participate for imaging by the time they were invited or had not matched any of the ASD participants and were thus dropped from the study. In addition, seven other TD participants met exclusionary criteria, which included a previous or current diagnosis of attention deficit hyperactivity disorder (2 participants), leukemia (1 participant), or MRI findings (4 participants). Among the potential ASD participants, two were excluded due to IQ < 80, three since they did not meet the threshold criteria for an ASD diagnosis, and one due to the presence of MRI contraindications. Two additional ASD participants requested to terminate the MRI procedure before any meaningful data could be acquired. Finally, two additional ASD data sets were not used in this study due to poor image quality. The remaining data sets (17 TD and 16 ASD participants) were included in the analyses (Table 1). Among the included ASD participants, 11 had participating parents that underwent the ADI-R.

### Magnetic resonance image acquisition

All MRI data were acquired on a 3T Trio MRI (Siemens Medical Solutions, Erlangen, Germany). Images were acquired using a body coil for transmission and a 12-channel array coil for reception. Diffusion imaging data were acquired using a twice-refocused diffusion-weighted echo planar imaging (EPI) sequence with a GRAPPA parallel imaging factor 2, and 24 reference lines. Between 55 and 60 slices were acquired using an isotropic voxel size of 2.3 × 2.3 × 2.3 mm^3^, TR = 8100 ms, and TE = 97 ms. Diffusion weighted imaging data were acquired for two b values (b = 1000 and 2000s/mm^2^) with 12 non-collinear encoding directions acquired for b = 1000 s/mm^2^, and 42 non-collinear encoding directions and for b = 2000s/mm^2^. Ten non-weighted diffusion images (b = 0 s/mm^2^) were also collected. Diffusion data acquisition was repeated twice. To correct for image distortions from B0 field inhomogeneities phase and magnitude field map images were acquired coplanar to the diffusion acquisition using the Siemens product sequence with echo-times of 8 ms and 10.46 ms.

In addition to the diffusion data, T1w images were acquired using a magnetization prepared rapid gradient-echo (MPRAGE) sequence and used in the atlas registration procedure for ROI analyses, and additionally for clinical evaluation of gross brain abnormalities in each subject. Two T1w protocols were used: a) an axial MPRAGE with a 192 × 256 × 188 matrix and a 1 mm^3^ isotropic voxel size was collected for 8 participants (2 ASD, 6 TD), and b) a MPRAGE with a 160 × 240 × 256 matrix and a 1 × 1 × 1.2 mm voxel size was collected for 25 participants (14 ASD, 11 TD). Eleven participants (9 ASD and 2 TD) had both T1w acquisitions with the rest of the participants having only one MPRAGE image.

### Image processing

Data were preprocessed and images were corrected for motion and distortions from eddy currents along with magnetic field inhomogeneities using in-house developed code in Matlab (Mathworks, Natick, Massachusetts), Interactive Data Language (IDL, Exelis Visual Information Solutions, Boulder, Colorado), and FMRIB Software Library (FSL4.1,http://www.fmrib.ox.ac.uk/fsl)^79^. Data pre-processing steps included: (1) correction of B0 field inhomogeneities using the field map and FSL fugue and prelude functions; (2) image smoothing with a three-dimensional Gaussian filter with σ = 1.2 mm; (3) visual inspection of images for signal dropouts, blurring from movement and removal of artefactual images, and; (4) adjustment of the encoding gradients’ matrix for rotations during the motion correction step. After image correction, diffusion and kurtosis tensors were calculated as previously described in the field^80^ and employed to calculate three-dimensional maps of MK and MD. Additionally, AK and RK maps were obtained to test for directional differences in kurtosis microstructural properties.

FreeSurfer (http://surfer.nmr.mgh.harvard.edu/; version 6.0) was used to construct the cortical surface of each participant based on the high-resolution T1w image. The FreeSurfer pipeline for surface construction, processed the images by segmentation of tissue types, tessellation of the grey/white matter junction, inflation of the folded surface and parcellation by the Desikan–Killiany atlas^81^. Each subject-specific cortically-labeled volume was then warped into dMRI space using FreeSurfer’s rigid-body transformation^81^. Mean diffusion metrics were obtained for each of the four regional cortical lobes and 68 cerebral cortex GM ROIs for each subject and used to conduct group comparisons.

To test if the use of two T1w acquisitions had an effect on dMRI metric values shown to be significantly different between groups (MK, RK, and MD) we conducted paired t-tests on data from 11 participants (9 ASD, 2 TD) that had both T1w acquisitions. For each of the 11 participants, image processing and registration to diffusion space was done separately for both T1w acquisitions and dMRI metrics were subsequently compared in the 68 ROIs delineated by the Desikan–Killiany atlas^82^. We found that T1w acquisition had no significant effect on RK or MK metrics (*p* > 0.05 for all comparisons) and minimal effect on MD metrics with no overlap between the ROIs affected by the T1W protocol and those shown to have significantly different MD in between-group comparisons (Supplementary Table 3).

### Statistical analyses

Primary analyses used analysis of covariance (ANCOVA) tests controlling for age to first compare diffusion metrics (MK, AK, RK and MD) across the four anatomically defined cortical lobes (frontal, parietal, temporal, and occipital), and then secondarily across the 68 cortical sub-lobar GM ROIs delineated by the Desikan–Killiany atlas^82^ using SPSS 20.0 (IBM, Armonk, NY). Although the two groups did not statistically differ in demographics (Table 1), DKI metrics have been shown to be sensitive to age^24,34–36^ and therefore age was included as a covariate in these analyses. Cohen’s d effect size was calculated to compare group means for each ROI analysis.

Pearson’s and Spearman’s correlations were used to explore the relationships between diffusion metrics that were found to show significant between-group differences (MK, RK, and MD) and ADI-R domain scores in ASD.

To account for multiple comparisons across the four cortical lobes and 68 cortical regions, we employed the Benjamini–Hochberg (BH) procedure^52^ to control the False Discovery Rate at 5%. Multiple comparison correction was applied first to (1) the 8 bilateral GM lobes, and then to (2) the 68 sub-cortical GM ROIs delineated by the Desikan–Killiany atlas. The Benjamini–Hochberg multiple comparison correction was performed on a single dMRI variable (MK, RK, AK and MD) at a time. Individual results were considered significant at BH q ≤ 0.05. Differences at *p* ≤ 0.05, uncorrected were considered to only reach trend-level and are listed in the tables and in the supplementary material.

## Supplementary information

Supplementary Tables.
