# Supplementary material for: Diffusion kurtosis imaging of gray matter in young adults with autism spectrum disorder
Source: Sci Rep. 2020 Dec 8;10:21465. doi: 10.1038/s41598-020-78486-w (PMC7722927; doi:10.1038/s41598-020-78486-w)
Supplement: Supplementary file 1 — Supplementary Tables. [file 41598_2020_78486_MOESM1_ESM.docx]

**Diffusion Kurtosis Imaging of Gray Matter in Young Adults with Autism Spectrum Disorder**

Faye McKenna, MS^1,3^, Laura Miles, PhD^1^, Jeffrey Donaldson, PhD^1,^ F. Xavier Castellanos^2,4^, MD, Mariana Lazar, PhD^1,3^

^1^Center for Biomedical Imaging, Department of Radiology, New York University School of Medicine, New York, NY, USA

^2^Department of Child and Adolescent Psychiatry, New York University School of Medicine, New York, NY, USA

^3^Sackler Institute of Graduate Biomedical Sciences, New York University School of Medicine, New York, NY, USA

^4^Nathan Kline Institute for Psychiatric Research, Orangeburg, NY, USA

**Supplementary Tables**

**Supplementary Table 1.** Spearman’s and Pearson’s correlations between RK and lifetime clinical symptoms as measured by ADI-R (n = 11). A higher score on the ADI-R indicates increased severity. All tests shown pass p<0.05, uncorrected, but none remained significant after correction, given the small sample with available data.

| **RK ROI** | **hemisphere** | **test** | **ADIR**  **Restrictive &repetitive behaviors**  R/p | **ADIR**  **Communication**  R/p | **ADIR**  **Social interaction**  R/p |
| --- | --- | --- | --- | --- | --- |
| Medial orbitofrontal | right | Spearman’s | -.77/.006 | – | -.64/.033 |
|  |  | Pearson’s | -.77/.006 | – | – |
| Medial orbitofrontal | left | Spearman’s | -.75/.008 | – | – |
|  |  | Pearson’s | -.74/.009 | – | – |
| Paracentral | left | Spearman’s | -.62/.044 | – | – |
|  |  | Pearson’s | – | – | – |
| Inferior Parietal | right | Spearman’s | -.60/.049 | – | – |
|  |  | Pearson’s | – | – | – |
| Inferior Temporal | left | Spearman’s | -.61/.046 | – | – |
|  |  | Pearson’s | -.64/.035 | – | – |
| Superior parietal | right | Spearman’s | – | -.72/.012 | – |
|  |  | Pearson’s | – | – | – |
| Parahippocampal | left | Spearman’s | -.62/.044 | – | – |
|  |  | Pearson’s | – | – | – |
| Caudal anterior cingulate | right | Spearman’s | -.71/.015 | – | – |
|  |  | Pearson’s | -.77/.006 | – | – |
| Insula | left | Spearman’s | -.61/.046 | – | – |
|  |  | Pearson’s | – | – | – |
| Isthmus cingulate | left | Spearman’s | -.65/.031 | – | – |
|  |  | Pearson’s | -.65/.032 | – | – |
| Posterior cingulate | right | Spearman’s | -.67/.025 | – | – |
|  |  | Pearson’s | – | – | – |
| Rostral anterior cingulate | right | Spearman’s | -.65/.031 | – | – |
|  |  | Pearson’s | -.63/.037 | – | – |

**Supplementary Table 2.** Spearman and Pearson’s correlations between MD and lifetime clinical symptoms as measured by ADI-R (n = 11). A higher score on the ADI-R indicates increased severity. All tests shown pass p<0.05, uncorrected, but none remained significant after correction, given the small sample with available data.

| **MD ROI** | **hemisphere** | **test** | **ADIR**  **Restrictive &repetitive behaviors**  R/p | **ADIR**  **Communication**  R/p |
| --- | --- | --- | --- | --- |
|  |  |  |  |  |
| Paracentral | right | Spearman’s | -.77/.006 | – |
|  |  | Pearson’s | -.73/.011 | – |
| Pars triangularis | right | Spearman’s | – | .69/.020 |
|  |  | Pearson’s | – | .70/.017 |
| Pars opercularis | right | Spearman’s | – | .64/.035 |
|  |  | Pearson’s | – | .67/.025 |
| Pars opercularis | left | Spearman’s | – | .74/.009 |
|  |  | Pearson’s | – | .62/.041 |
| Precentral | left | Spearman’s | -.62/.046 | – |
|  |  | Pearson’s | – | – |
| Rostral middle frontal | right | Spearman’s | – | .66/.026 |
|  |  | Pearson’s | – | – |
| Postcentral | left | Spearman’s | -.64/.034 | – |
|  |  | Pearson’s | – | – |
| Precuneus | right | Spearman’s | -.64/.034 | – |
|  |  | Pearson’s | – | – |
| Supramarginal | left | Spearman’s | -.71/.014 | – |
|  |  | Pearson’s | – | – |
| Caudal anterior cingulate | left | Spearman’s | – | .71/.014 |
|  |  | Pearson’s | – | .77/.006 |
| Isthmus cingulate | right | Spearman’s | -.74/.009 | – |
|  |  | Pearson’s | -.73/.01 | – |
| Posterior cingulate | right | Spearman’s | -.71/.014 | – |
|  |  | Pearson’s | – | – |
| Rostral anterior cingulate | left | Spearman’s | – | .64/.034 |
|  |  | Pearson’s | – | .69/.018 |

**Supplementary Table 3.** Paired T-test in 11 participants (9 ASD, 2 TD) comparing the two protocol’s T1-weighted images effect on the 68 Desikan-Killiany atlas-based diffusion metrics. No significant differences were found between the two protocol’s T1-weighted atlas-based ROI analysis of MK or RK metrics. Minimal effect was found on MD metrics in non-overlapping ROIs shown to be significant by between-group tests.

| **Region** | | **Hemisphere** | **MK** protocol 1 | **MK** protocol 2 | **P** value | **RK** protocol 1 | **RK**  protocol 2 | **P** value | **MD** protocol 1  **(**µm^2^/ms) | **MD**  protocol 2  **(**µm^2^/ms) | **P** value |
| --- | --- | --- | --- | --- | --- | --- | --- | --- | --- | --- | --- |
| **Frontal Lobe** | Caudal middle frontal | right | .76+/-.03 | 75+/-.03 | .36 | .78+/-.05 | .76+/-.02 | .31 | 1.19+/-.09 | 1.26+/-.16 | .10 |
|  | Caudal middle frontal | left | .75+/-.03 | .75+/-.03 | .94 | .76+/-.04 | .75+/-.05 | .85 | 1.21+/-.11 | 1.26+/-.15 | .21 |
|  | Frontal pole | right | .71+/-.07 | .70+/-.07 | .94 | .70+/-.09 | .68+/-.08 | .72 | 1.24+/-.21 | 1.26+/-.18 | .62 |
|  | Frontal pole | left | .71+/-.05 | .71+/-.06 | .68 | .69+/-.04 | .70+/-.07 | .87 | 1.26+/-.28 | 1.3+/-.22 | .40 |
|  | Lateral orbitofrontal | right | .68+/-.03 | .66+/-.03 | .19 | .65+/-.05 | .63+/-.05 | .41 | 1.15+/-.08 | 1.17+/-.10 | .54 |
|  | Lateral orbitofrontal | left | .68+/-.03 | .68+/-.04 | .58 | .66+/-.03 | .65+/-.06 | .79 | 1.17+/-.09 | 1.17+/-.07 | .92 |
|  | Medial orbitofrontal | right | .62+/-.04 | .61+/-.03 | .50 | .58+/-.04 | .58+/-.03 | .46 | 1.19+/-.12 | 1.22+/-.10 | .43 |
|  | Medial orbitofrontal | left | .63+/-.05 | .63+/-.03 | .80 | .60+/-.05 | .60+/-.03 | .92 | 1.22+/-.10 | 1.16+/-.12 | .03 |
|  | Paracentral | right | .73+/-.04 | .74+/-.04 | .38 | .73+/-.05 | .74+/-.06 | .43 | 1.25+/-.12 | 1.26+/-.21 | .91 |
|  | Paracentral | left | .75+/-.04 | .74+/-.05 | .43 | .76+/-.04 | .74+/-.06 | .43 | 1.31+/-.18 | 1.41+/-.15 | .06 |
|  | Pars opercularis | right | .71+/-.01 | .72+/-.01 | .32 | .72+/-.03 | .74+/-.03 | .30 | 1.13+/-.09 | 1.18+/-.10 | .003 |
|  | Pars opercularis | left | .72+/-.03 | .72+/-.03 | .79 | .74+/-.03 | .73+/-.04 | .91 | 1.18+/-.07 | 1.18+/-.06 | .72 |
|  | Pars orbitalis | right | .72+/-.03 | .71+/-.04 | .64 | .70+/-.04 | .70+/-.05 | .62 | 1.09+/-.08 | 1.14+/-.07 | .001 |
|  | Pars orbitalis | left | .70+/-.04 | .70+/-.04 | .54 | .69+/-.05 | .68+/-.06 | .72 | 1.15+/-.10 | 1.17+/-.13 | .66 |
|  | Pars triangularis | right | .75+/-.03 | .74+/-.03 | .31 | .75+/-.04 | .74+/-.04 | .56 | 1.14+/-.09 | 1.2+/-.14 | .08 |
|  | Pars triangularis | left | .73+/-.03 | .73+/-.03 | .67 | .74+/-.03 | .73+/-.04 | .88 | 1.2+/-.09 | 1.2+/-.09 | .97 |
|  | Precentral | right | .78+/-.02 | .76+/-.03 | .08 | .81+/-.03 | .78+/-.04 | .15 | 1.26+/-.11 | 1.36+/-.19 | .02 |
|  | Precentral | left | .77+/-.03 | .76+/-.03 | .41 | .71+/-.04 | .71+/-.05 | .55 | 1.25+/-.10 | 1.31+/-.18 | .17 |
|  | Rostral middle frontal | right | .73+/-.03 | .72+/-.03 | 1.0 | .74+/-.04 | .73+/-.04 | .49 | 1.15+/-.11 | 1.23+/-.17 | .04 |
|  | Rostral middle frontal | left | .71+/-.02 | .71+/-.03 | .52 | .72+/-.03 | .72+/-.04 | .90 | 1.18+/-.10 | 1.19+/-.09 | .88 |
|  | Superior frontal | right | .70+/-.03 | .70+/-.03 | .82 | .69+/-.04 | .68+/-.04 | .85 | 1.21+/-.09 | 1.23+/-.11 | .34 |
|  | Superior frontal | left | .71+/-.02 | .70+/-.02 | .59 | .70+/-.04 | .70+/-.04 | .75 | 1.22+/-.10 | 1.26+/-.12 | .22 |
| **Parietal Lobe** | Inferior parietal | right | .74+/-.02 | .74+/-.02 | .98 | .75+/-.03 | .74+/-.03 | .29 | 1.08+/-.07 | 1.14+/-.12 | .03 |
|  | Inferior parietal | left | .73+/-.02 | .74+/-.02 | .88 | .74+/-.05 | .74+/-.05 | .99 | 1.09+/-.09 | 1.16+/-.17 | .05 |
|  | Postcentral | right | .76+/-.02 | .74+/-.02 | .25 | .78+/-.03 | .76+/-.04 | .26 | 1.36+/-.12 | 1.44+/-.14 | .02 |
|  | Postcentral | left | .76+/-.02 | .74+/-.03 | .32 | .77+/-.03 | .75+/-.04 | .32 | 1.34+/-.10 | 1.4+/-.15 | .17 |
|  | Precuneus | right | .70+/-.03 | .70+/-.03 | .77 | .71+/-.05 | .71+/-.04 | .78 | 1.17+/-.08 | 1.16+/-.11 | .62 |
|  | Precuneus | left | .70+/-.03 | .70+/-.04 | .66 | .70+/-.04 | .70+/-.05 | .75 | 1.19+/-.12 | 1.28+/-.12 | .003 |
|  | Superior parietal | right | .76+/-.02 | .74+/-.04 | .12 | .79+/-.02 | .78+/-.04 | .25 | 1.30+/-.13 | 1.45+/-.24 | .03 |
|  | Superior parietal | left | .75+/-.02 | .74+/-.03 | .23 | .77+/-.03 | .75+/-.04 | .24 | 1.29+/-.13 | 1.42+/-.29 | .06 |
|  | Supramarginal | right | .73+/-.02 | .73+/-.02 | .75 | .75+/-.02 | .74+/-.02 | .84 | 1.18+/-.08 | 1.24+/-.10 | .02 |
|  | Supramarginal | left | .71+/-.02 | .71+/-.02 | .88 | .71+/-.04 | .71+/-.04 | .90 | 1.18+/-.09 | 1.20+/-.12 | .34 |
| **Temporal Lobe** | Bankssts | right | .74+/-.03 | .74+/-.03 | .80 | .76+/-.01 | .75+/-.01 | .92 | 1.01+/-.05 | 1.02+/-.05 | .54 |
|  | Bankssts | left | .72+/-.04 | .73+/-.05 | .54 | .72+/-.01 | .74+/-.02 | .52 | 1.02+/-.06 | 1.01+/-.07 | .38 |
|  | Entorhinal | right | .74+/-.03 | .74+/-.03 | .97 | .65+/-.02 | .62+/-.03 | .85 | 1.53+/-.38 | 1.53+/-.23 | .99 |
|  | Entorhinal | left | .66+/-.06 | .67+/-.05 | .55 | .63+/-.02 | .62+/-.01 | .79 | 1.35+/-.24 | 1.45+/-.27 | .31 |
|  | Fusiform | right | .68+/-.05 | .68+/-.06 | .98 | .64+/-.05 | .64+/-.07 | .96 | 1.03+/-.08 | 1.00+/-.06 | .10 |
|  | Fusiform | left | .68+/-.03 | .67+/-.05 | .70 | .63+/-.05 | .64+/-.07 | .69 | 1.03+/-.06 | 1.01+/-.06 | .50 |
|  | Parahippocampal | right | .67+/-.05 | .68+/-.06 | .62 | .65+/-.06 | .66+/-.07 | .40 | 1.33+/-.16 | 1.24+/-.15 | .23 |
|  | Parahippocampal | left | .65+/-.05 | .65+/-.05 | 1.0 | .62+/-.02 | .63+/-.02 | .69 | 1.33+/-.16 | 1.34+/-.23 | .89 |
|  | Inferior temporal | right | .64+/-.05 | .65+/-.06 | .77 | .59+/-.04 | .58+/-.06 | .94 | 1.00+/-.06 | 0.99+/-.06 | .40 |
|  | Inferior temporal | left | .78+/-.04 | .86+/-.05 | .72 | .58+/-.05 | .59+/-.07 | .65 | 1.02+/-.05 | 1.01+/-.05 | .82 |
|  | Middle temporal | right | .68+/-.04 | .69+/-.05 | .36 | .65+/-.04 | .66+/-.03 | .43 | 1.02+/-.05 | 1.05+/-.09 | .09 |
|  | Middle temporal | left | .67+/-.03 | .67+/-.04 | .77 | .64+/-.04 | .64+/-.05 | .74 | 1.05+/-.07 | 1.05+/-.05 | .99 |
|  | Superior temporal | right | .71+/-.02 | .71+/-.02 | .87 | .71+/-.03 | .70+/-.03 | .55 | 1.15+/-.06 | 1.18+/-.09 | .28 |
|  | Superior temporal | left | .70+/-.02 | .70+/-.02 | .80 | .69+/-.04 | .69+/-.04 | .94 | 1.16+/-.06 | 1.20+/-.08 | .09 |
|  | Temporal pole | right | .69+/-.05 | .69+/-.06 | .91 | .67+/-.08 | .66+/-.09 | .88 | 1.15+/-.12 | 1.25+/-.16 | .11 |
|  | Temporal pole | left | .67+/-.05 | .68+/-.06 | .42 | .67+/-.08 | .66+/-.08 | .73 | 1.22+/-.16 | 1.31+/-.25 | .16 |
|  | Transverse temporal | right | .78+/-.02 | .77+/-.04 | .60 | .79+/-.03 | .79+/-.05 | .61 | 1.25+/-.08 | 1.28+/-.15 | .49 |
|  | Transverse temporal | left | .78+/-.02 | .77+/-.03 | .19 | .79+/-.05 | .77+/-.05 | .40 | 1.19+/-.09 | 1.25+/-.11 | .09 |
|  | Cuneus | right | .78+/-.02 | .77+/-.03 | .42 | .78+/-.03 | .77+/-.06 | .22 | 1.23+/-.11 | 1.28+/-.19 | .33 |
| **Occipital Lobe** | Cuneus | left | .77+/-.03 | .76+/-.04 | .33 | .78+/-.03 | .76+/-.05 | .38 | 1.22+/-.12 | 1.33+/-.14 | .01 |
|  | Lateral occipital | right | .79+/-.04 | .79+/-.04 | .66 | .77+/-.05 | .77+/-.04 | .22 | 1.05+/-.06 | 1.07+/-.09 | .22 |
|  | Lateral occipital | left | .79+/-.04 | .79+/-.03 | .45 | .77+/-.05 | .77+/-.04 | .94 | 1.04+/-.06 | 1.08+/-.09 | .05 |
|  | Lingual | right | .74+/-.03 | .73+/-.04 | .62 | .73+/-.04 | .74+/-.05 | .50 | 1.28+/-.10 | 1.2+/-.10 | .08 |
|  | Lingual | left | .74+/-.02 | .73+/-.04 | .77 | .73+/-.05 | .72+/-.04 | .75 | 1.22+/-.11 | 1.25+/-.15 | .59 |
|  | Pericalcarine | right | .81+/-.03 | .80+/-.02 | .70 | .83+/-.04 | .85+/-.03 | .35 | 1.23+/-.11 | 1.17+/-.10 | .05 |
|  | Pericalcarine | left | .81+/-.03 | .80+/-.03 | .71 | .83+/-.05 | .81+/-.04 | .60 | 1.23+/-.12 | 1.22+/-.10 | .90 |
| **Other** | Caudal anterior cingulate | right | .65+/-.04 | .66+/-.05 | .55 | .63+/-.05 | .62+/-.07 | .73 | 1.08+/-.09 | 1.06+/-.06 | .41 |
|  | Caudal anterior cingulate | left | .63+/-.04 | .62+/-.04 | .70 | .66+/-.04 | .68+/-.10 | .59 | 1.07+/-.09 | 1.1+/-.10 | .33 |
|  | Insula | right | .68+/-.04 | .67+/-.04 | .77 | .70+/-.05 | .68+/-.05 | .53 | 1.09+/-.06 | 1.16+/-.10 | .04 |
|  | Insula | left | .67+/-.04 | .67+/-.03 | .66 | .68+/-.04 | .69+/-.05 | .39 | 1.11+/-.08 | 1.09+/-.06 | .37 |
|  | Isthmus cingulate | right | .67+/-.06 | .67+/-.06 | .89 | .69+/-.07 | .69+/-.06 | 1.0 | 1.16+/-.08 | 1.07+/-.09 | .05 |
|  | Isthmus cingulate | left | .68+/-.06 | .67+/-.06 | .69 | .70+/-.05 | .69+/-.07 | .79 | 1.18+/-.17 | 1.25+/-.20 | .36 |
|  | Posterior cingulate | right | .66+/-.05 | .66+/-.05 | .94 | .68+/-.05 | .67+/-.06 | .62 | 1.07+/-.10 | 1.07+/-.11 | .93 |
|  | Posterior cingulate | left | .69+/-.05 | .68+/-.04 | .79 | .70+/-.06 | .68+/-.05 | .40 | 1.09+/-.09 | 1.14+/-.11 | .02 |
|  | Rostral anterior cingulate | right | .62+/-.06 | .60+/-.04 | .74 | .60+/-.06 | .59+/-.05 | .77 | 1.08+/-.08 | 1.08+/-.06 | .88 |
|  | Rostral anterior cingulate | left | .62+/-.05 | .62+/-.04 | .98 | .61+/-.06 | .61+/-.05 | .85 | 1.09+/-.08 | 1.11+/-.09 | .44 |
